# Cell surface GRP78 promotes tumor cell histone acetylation through metabolic reprogramming: a mechanism which modulates the Warburg effect

**DOI:** 10.18632/oncotarget.22431

**Published:** 2017-11-14

**Authors:** Udhayakumar Gopal, Salvatore V. Pizzo

**Affiliations:** ^1^ Department of Pathology, Duke University Medical Center, Durham, NC, USA

**Keywords:** Cell Surface GRP78, α_2_M^*^, histone acetylation, acetyl-CoA, metabolism

## Abstract

Acetyl coenzyme A (acetyl-CoA) is essential for histone acetylation, to promote cell proliferation by regulating gene expression. However, the underlying mechanism(s) governing acetylation remains poorly understood. Activated α_2_-Macroglobulin (α_2_M^*^) signals through tumor Cell Surface GRP78 (CS-GRP78) to regulate tumor cell proliferation through multiple signaling pathway. Here, we demonstrate that the α_2_M^*^/CS-GRP78 axis regulates acetyl-CoA synthesis and thus functions as an epigenetic modulator by enhancing histone acetylation in cancer cells. α_2_M^*^/CS-GRP78 signaling induces and activates glucose-dependent ATP-citrate lyase (ACLY) and promotes acetate-dependent Acetyl-CoA Synthetase (ACSS1) expression by regulating AKT pathways to acetylate histones and other proteins. Further, we show that acetate itself regulates ACLY and ACSS1 expression through a feedback loop in an AKT-dependent manner. These studies demonstrate that α_2_M^*^/CS-GRP78 signaling is a central mechanism for integrating glucose and acetate-dependent signaling to induce histone acetylation. More importantly, targeting the α_2_M^*^/CS-GRP78 axis with C38 Monoclonal antibody (Mab) abrogates acetate-induced acetylation of histones and proteins essential for proliferation and survival under hypoxic stress. Furthermore, C38 Mab significantly reduced glucose uptake and lactate consumption which definitively suggests the role of aerobic glycolysis. Collectively, besides its ability to induce fatty acid synthesis, our study reveals a new mechanism of epigenetic regulation by the α_2_M^*^/CS-GRP78 axis to increase histone acetylation and promote cell survival under unfavorable condition. Therefore CS-GRP78 might be effectively employed to target the metabolic vulnerability of a wide spectrum of tumors and C38 Mab represents such a potential therapeutic agent.

## INTRODUCTION

Metabolic reprogramming is an essential mechanism in cancer cell biology [1, 2]. Acetyl-CoA, as a central metabolic intermediate, is widely used in macromolecule biosynthesis and energy production to support cancer cell proliferation. As a donor of acetyl group, acetyl-CoA is also dynamically associated with acetylation to modulate protein functions. Therefore, maintenance of the cellular acetyl-CoA pools is essential for regulating various cellular processes. Acetyl-CoA is compartmentalized into mitochondrial and nuclear/cytoplasmic pools [3]. Production of nuclear/cytoplasmic acetyl-CoA from glucose, glutamine, or fatty acids depends on export of citrate from the mitochondria, where it is cleaved by ACLY to generate acetyl-CoA and oxaloacetate. ACLY is required for cell proliferation and cancer cell survival and there is interest in the development of ACLY inhibitors with some showing potential for inhibiting tumor growth [4–6]. Recent studies demonstrate that cancer cells avidly capture acetate as their alternative carbon source to glucose to maintain cellular acetyl-CoA pools under stressed conditions [7–13]. How cancer cells utilize glucose and acetate under nutrient limitation in such an efficient manner remains unclear. Acetate is mainly acquired from the diet and gut microbiota [14, 15], but also be generated from ethanol metabolism or deacetylation processes. The function of acetate has long been overlooked perhaps due to its relatively low physiological concentration, 0.2-0.3 mM, in blood [16]. Various human cancers show enhanced acetate uptake in [^11^C]-acetate PET studies [17–20]. These findings suggest that cancer cells utilize acetate to supply acetyl-CoA through acetyl-CoA synthetases (ACSS1/ACSS2). Together ACLY and ACSS1 are the primary enzymatic sources of acetyl-CoA, but relatively little is known regarding the mechanism and regulation of their gene expression. The interplay between ACLY and ACSS1 in the control of acetyl-CoA metabolism is still obscure.

Histone acetylation is intimately coordinated with the cellular acetyl-CoA pools in response to the existing metabolic state. Several studies demonstrate that histone acetylation is highly sensitive to the availability of acetyl-CoA in organisms from yeast to human [21–23]. Thus the acetyl-CoA flux dynamically regulates the gene expression profile by modulating histone acetylation state. Epigenetic alteration plays a key role in enabling malignant transformation and tumor proliferation but the underlying mechanisms are not fully understood. Understanding how cancer cells harness cellular metabolism and its metabolites for their survival may yield insights into cancer pathogenesis and the mechanism that tumor cells use to survive under metabolic limitations.

Metabolic changes in cancer cells are typically mediated by activation of oncogenes and/or loss of tumor suppressors [24, 25]. The Glucose Regulated Protein 78 (GRP78), a major chaperone in the endoplasmic reticulum, is expressed on the cell surface of stressed cancer cells, where it regulates critical oncogenic signaling pathways and has emerged as a target for anti-cancer therapy [26–32]. Cell Surface GRP78 (CS-GRP78) acts as a multifunctional receptor that affects both cell proliferation and viability. CS-GRP78 functions as a receptor for the activated form of the plasma proteinase inhibitor α_2_-Macroglobulin (α_2_M^*^) and it is involved in the development of metastatic prostate cancer [26, 30, 33]. Activation of CS-GRP78 by α_2_M^*^ requires ligation of the GRP78 primary amino acid sequence (Leu^98^-Leu^115^) [26, 34]. Notably, we and others have shown that the α_2_M^*^/CS-GRP78 axis functions as an upstream regulator of PI 3-kinase/AKT oncogenic signaling pathways to regulate cell proliferation, survival, gene transcription, metastasis and chemoresistance [26–28, 33]. Furthermore, α_2_M^*^/CS-GRP78 axis is required for the transcriptional activation of subset of c-Myc target genes and cell transformation [26]. Importantly, targeting CS-GRP78 by C38 or other specific monoclonal antibodies inhibits tumor growth in murine xenograft models of various tumors [28, 31, 35]. In addition, recently we reported that the α_2_M^*^/CS-GRP78 axis regulates metabolic alterations to enhance aerobic glycolysis and induce fatty acid synthesis in prostate cancer cells [36]. However, it is not known whether the α_2_M^*^/CS-GRP78 axis contributes to histone acetylation by regulating metabolic reprogramming. These observations led us to hypothesis that the α_2_M^*^/CS-GRP78 axis induces a metabolic adaptation through modulating acetyl-CoA production and histone acetylation in cancer cells. Consistent with this idea, we found that the α_2_M^*^/CS-GRP78 axis predominately activates expression of ACLY and ACSS1 through AKT signaling which in turn upregulates the acetyl-CoA production and histone acetylation. Our current studies demonstrate the mechanisms by which the α_2_M^*^/CS-GRP78 axis promotes the Warburg effect in cancer cells. Beyond the role as a pro-proliferative signaling mechanism, our findings highlight an epigenetic role for the α_2_M^*^/CS-GRP78 axis in metabolic adaptation of cancer cells.

## RESULTS

### α_2_M^*^/CS-GRP78 signaling regulates acetyl-CoA production and histone acetylation

**Figure 1:**
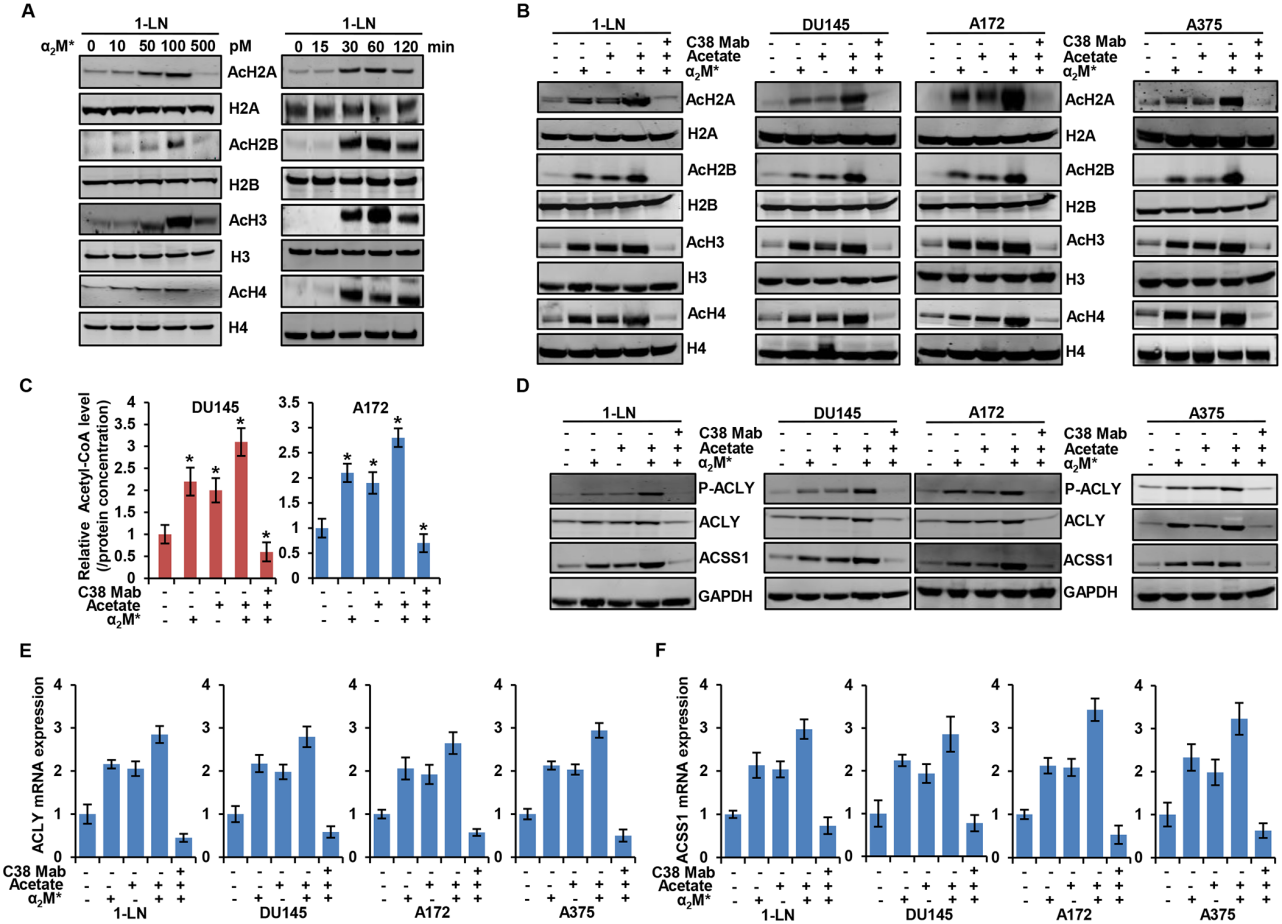
α_2_M^*^ signals through CS-GRP78 to regulate acetyl-CoA production and histone acetylation **(A)** Immunoblot analysis showing histone acetylation levels in 1-LN prostate cancer cell line after stimulated with α_2_M^*^ at the indicated dose response for 30 min (left panel) and α_2_M^*^ (100 pM) for the indicated time points. **(B)** Immunoblot analysis of the indicated cancer cell lines treated with C38 Mab (50 μg) for 6 h and then stimulated with α_2_M^*^ (100 pM) for 30 min and acetate (5 mM) for 4 h alone or in combination. **(C)** Total cellular concentration of acetyl-CoA was measured in indicated cell lines treated with C38 Mab (50 μg) for 6 h and then stimulated with α_2_M^*^ (100 pM) for 30 min and acetate (5mM) for 4 h alone or in combination. mean ± SEM of triplicates. **(D)** Immunoblot analysis of the indicated cancer cell lines treated with C38 Mab (50 μg) for 6 h and then stimulated with α_2_M^*^ (100 pM) for 30 min and acetate (5mM) for 4 h alone or in combination. **(E-F)** Indicated cancer cell lines were treated with C38 Mab (50 μg) for 6 h and then stimulated with α_2_M^*^ (100 pM) for 30 min and acetate (5 mM) for 4 h alone or in combination to quantify the transcript level of ACLY and ACSS1 genes. ^*^, *p* values ≤ 0.05. Error bar represent S.D.

To examine how tumor cells regulate cellular metabolism, we analyzed the effects on intermediary metabolites by α_2_M^*^/CS-GRP78 signaling. In 1-LN prostate cancer cells, α_2_M^*^ induced significant increase of H2A, H2B, H3, and H4 acetylation in a dose- and time-dependent manner (Figure 1A). α_2_M^*^ signals through CS-GRP78 to regulate cell proliferation which was abrogated by treating with C38 Mab and we now hypothesize that regulation of histone acetylation by α_2_M^*^ may account for these observations [26, 31]. To characterize the manner of epigenetic regulation by α_2_M^*^ signaling, we treated a prostate cancer, glioblastoma, and melanoma cancer cell lines with C38 Mab and then stimulated with α_2_M^*^ and acetate alone or in combination. α_2_M^*^ rapidly increased H2A, H2B, H3 and H4 acetylation and acetate synergistically increased the effect. Targeting CS-GRP78 with our previously described C38 Mab [26] abrogates the α_2_M^*^- and acetate-induced increase in acetylation (Figure 1B). Collectively, these data imply that α_2_M^*^/CS-GRP78 signaling regulates histone acetylation. Acetyl-CoA is one of the intermediate metabolites produced from glucose and acetate to regulate cell proliferation [21]. We thus hypothesized that the α_2_M^*^/CS-GRP78 signaling axis regulates histone acetylation through acetyl-CoA production. We measured the level of acetyl-CoA in DU145 and A172 cell lines treated with α_2_M^*^ and acetate alone or in combination and determined the effect of C38 Mab. Acetyl-CoA production increased in α_2_M^*^ stimulation alone without acetate addition yet each remains responsive to C38 Mab (Figure 1C).

In mammals, two primary enzymes are involved in acetyl-CoA production from acetate, cytosolic ACSS2 and its mitochondrial homologue ACSS1 [16]. Recent studies also highlight that ACSS1 and ACSS2 are functionally redundant [37–39], and in our current studies, we primarily focused on the ACSS1. Previous studies also show that acetyl-CoA is produced from glucose by the enzyme adenosine triphosphate (ATP)-citrate lyase (ACLY) which generates acetyl-CoA from mitochondria-derived citrate [5]. To dissect which enzyme is responsible for mediating α_2_M^*^-induced acetyl-CoA production and histone acetylation, we stimulated the panel of cancer cell lines with α_2_M^*^ and acetate either alone or in combination in the presence and absence of C38 Mab. α_2_M^*^- and acetate- synergistically increased phosphorylation of ACLY and expression of ACLY and ACSS1 whereas targeting CS-GRP78 suppressed this effect (Figure 1D). In our previous studies we identified the GRP78 primary amino acid sequence LIGRTWNDPSVQQDIKFL (Leu^98^-Leu^115^) as the putative binding site for α_2_M^*^, which is essential for triggering downstream signaling [26, 34]. We next studied the specificity of CS-GRP78 signaling by stimulating the various cancer cell lines with α_2_M^*^ in the presence of scrambled (Scr) or GRP78 (Leu^98^-Leu^115^), peptides. GRP78 peptide decreased α_2_M^*^-dependent phosphorylation of ACLY and it also suppressed ACLY and ACSS1 induction. In contrast, the Scr peptide did not affect α_2_M^*^-mediated ACLY and ACSS1 induction or phosphorylation of ACLY (Supplementary Figure 1A). These results further demonstrate that α_2_M^*^ signals specifically through the GRP78 (Leu^98^-Leu^115^) binding site to induce ACLY and ACSS1 expression. We further investigated whether α_2_M^*^- and acetate-induced ACLY and ACSS1 expression is associated with gene transcription. We treated the various cancer cell lines with C38 Mab and then exposed them to α_2_M^*^ and acetate either alone or in combination. α_2_M^*^ and acetate drastically increases mRNA expression of ACLY and ACSS1. We found increased mRNA expression of ACLY and ACSS1 in α_2_M^*^ stimulation without acetate addition, but the acetate effects are also responsive to C38 Mab presumably dependent on CS-GRP78 mediated signaling (Figure 1E and 1F). These results demonstrate that α_2_M^*^ signals through CS-GRP78 to promote the expression of ACLY and ACSS1 at the transcript level.

### α_2_M^*^/CS-GRP78 signaling promotes histone acetylation in an AKT-dependent manner

**Figure 2:**
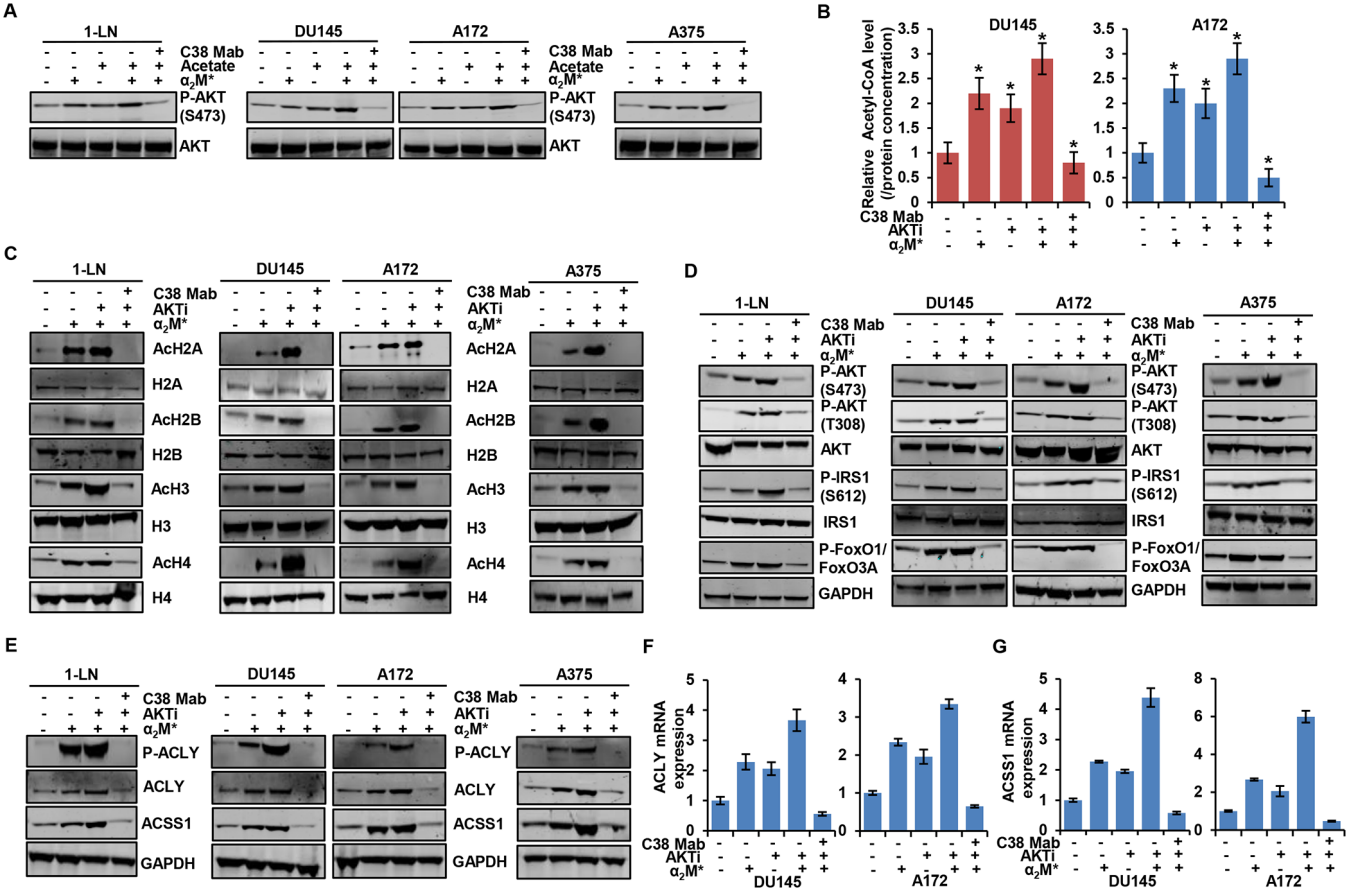
α_2_M^*^/CS-GRP78 axis induces histone acetylation via an AKT signaling pathway **(A)** Immunoblot analysis of the indicated cancer cell lines treated with C38 Mab (50 μg) for 6 h and then stimulated with α_2_M^*^ (100 pM) for 30 min and acetate (5 mM) for 4 h alone or in combination. **(B)** The total cellular concentration of acetyl-CoA was measured from the indicated cell lines stimulated with α_2_M^*^ (100 pM) for 30 min in the absence and presence of AKTi (GSK690693, 5 μM/L) for 16 h or C38 Mab (50 μg) for 6 h. mean ± SEM of triplicates. **(C)** Immunoblot analysis of the indicated cancer cell lines stimulated with α_2_M^*^ (100 pM) for 30 min in the absence and presence of AKTi (GSK690693, 5 μM/L) for 16 h or C38 Mab (50 μg) for 6 h. **(D)** Immunoblot analysis of AKT activation in indicated cancer cell lines stimulated with α_2_M^*^ (100 pM) for 30 min in the absence and presence of AKTi (GSK690693, 5 μM/L) for 16 h or C38 Mab (50 μg) for 6 h. **(E)** Immunoblot analysis of the indicated cancer cell lines stimulated with α_2_M^*^ (100 pM/L) for 30 min in the absence and presence of AKTi (GSK690693, 5 μM/L) for 16 h or C38 Mab (50 μg) for 6 h. **(F-G)** Indicated cancer cell lines treated with AKTi (GSK690693, 5 μM/L) for 16 h or C38 Mab (50 μg) for 6 h alone or in combination and then stimulated with α_2_M^*^ (100 pM) for 30 min to quantify the transcript level of ACLY and ACSS1 genes. ^*^, *p* values ≤ 0.05. Error bar represent S.D.

We next examined signaling pathways downstream of the α_2_M^*^/CS-GRP78 axis to identify those responsible for elevating histone acetylation in cancer cells. Previous studies demonstrate that CS-GRP78 is a potent regulator of the PI 3-kinase/AKT signaling pathways to promote tumor proliferation and prolong survival [26–28, 33]. Moreover, this pathway is a key determinant of histone acetylation in tumor cells by modulating metabolic reprogramming [22, 40]. To test whether α_2_M^*^/CS-GRP78 signaling regulates AKT activation to modulate histone acetylation, we treated the various cancer cell lines with C38 Mab and then stimulated with α_2_M^*^ and acetate either alone or in combination. As expected we observed that α_2_M^*^ induced the phosphorylation of AKT S473; surprisingly acetate augmenting the phosphorylation of AKT. These studies are consistent with previous findings which report that acetate promotes mTORC2 signaling [41]. On the other hand, C38 Mab suppressed both α_2_M^*^- and augmented acetate-induced phosphorylation of AKT (Figure 2A). To determine whether CS-GRP78-mediated AKT activation regulates acetyl-CoA, we treated DU145 and A172 cell lines with C38 Mab, the pan-AKT inhibitor GSK690693 (AKTi) alone or in combination and then stimulated with α_2_M^*^. α_2_M^*^ induced acetyl-CoA in cancer cells, but surprisingly, AKTi also elevates acetyl-CoA which is discrepant from two previous studies [22, 41]. This may reflect the nature of this ATP-competitive AKT inhibitor which induces negative feedback activation of the PI 3-kinase/AKT pathway as previously shown [42, 43]. Moreover, C38 Mab significantly reduced α_2_M^*^- and AKTi-induced acetyl-CoA production showing that targeting CS-GRP78 reverses the activity of this AKT inhibitor (Figure 2B). To determine whether AKTi-induced acetyl-CoA synthesis affects histone acetylation we treated the various cancer cell lines with C38 Mab and AKTi alone or in combination and then stimulated with α_2_M^*^. Exogenous α_2_M^*^ rapidly increased H2A, H2B, H3 and H4 acetylation as does AKTi treatment. Further, AKTi augments acetate-induced histone acetylation. However, C38 Mab suppressed α_2_M^*^ and AKTi-induced histone acetylation (Figure 2C and Supplementary Figure 2A). Collectively, these data suggest that CS-GRP78 regulates acetyl-CoA metabolism and histone acetylation in an AKT-dependent manner.

To determine whether AKTi modulates cell surface GRP78 expression, we treated the various cancer cell lines with AKTi and did not observe any significant changes in CS-GRP78 expression (Supplementary Figure 2B). We further investigated whether CS-GRP78 plays an important regulatory role in the ability of AKT inhibitors to modulate acetyl-CoA and histone acetylation. We treated the various cancer cell lines with C38 Mab and AKTi and then stimulated with α_2_M^*^. AKTi and C38 Mab completely blocked α_2_M^*^-induced phosphorylation of two well-known AKT substrates, GSK3β and PRAS40, showing the effectiveness of this compound (Supplementary Figure 2C). We also observed that AKTi augmented α_2_M^*^-induced phosphorylation of ERK1/2 similar to the previous findings that inhibition of the PI 3-kinase pathway leads to activation of the mitogen activated protein kinase pathway [42]. Importantly, C38 Mab effectively blocked the AKTi-induced ERK pathway (Supplementary Figure 2C). In agreement with previous reports [42, 43] that AKT inhibitors cause the paradoxical hyperphosphorylation of AKT at its two regulatory sites (Thr^308^ and Ser^473^) and IRS1 (Ser^612^) whereas C38 Mab blocked this event. These results demonstrate the role of CS-GRP78 plays in conjunction with this AKT inhibitor (Figure 2D). In addition, C38 Mab blocked AKTi-mediated negative feedback activation of FOXO transcription factor (Figure 2D). The addition of C38 Mab reduced α_2_M^*^- and AKTi-induced phosphorylation of ACLY and upregulation of ACLY and ACSS1 expression at both the protein and transcript levels (Figure 2E, and 2G). Further, AKTi also augments acetate-induced phosphorylation of ACLY and upregulation of ACLY and ACSS1 expression (Supplementary Figure 2D). These data indicate the mechanism by which AKTi augments ACLY and ACSS1 expression for acetyl-CoA production and histone acetylation. When we employed another non-ATP-competitive AKT inhibitor (AKTi1/2) it blocked α_2_M^*^ and acetate induced AKT phosphorylation, acetyl-CoA production, histone acetylation, phosphorylation of ACLY and upregulation of ACLY and ACSS1 expression at both the protein and transcript level (Supplementary Figure 3A-3E). Therefore, we hypothesize that targeting CS-GRP78 is more effective than employing ATP competitive-AKT inhibitors alone. These studies clearly demonstrate that α_2_M^*^/CS-GRP78 signaling regulates the expression of both ACSS1 and ACLY enzymes through AKT signaling which is essential for acetyl-CoA production and histone acetylation.

### ACLY mediates histone acetylation in response to the α_2_M^*^/CS-GRP78 signaling

**Figure 3:**
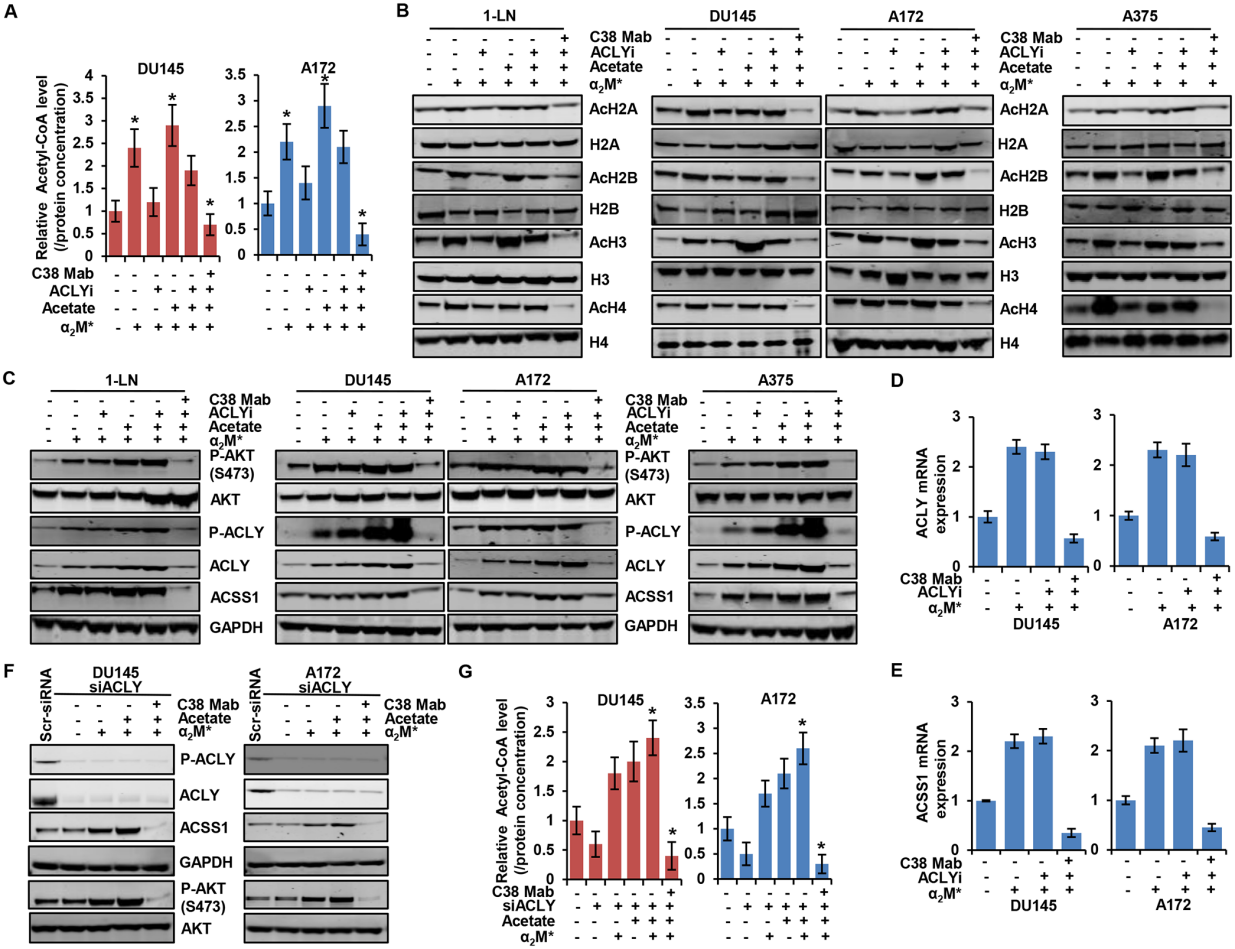
α_2_M^*^/CS-GRP78 controls ACLY expression to regulate histone acetylation **(A)** The total cellular concentration of acetyl-CoA was measured in the indicated cell lines stimulated with either α_2_M^*^ (100 pM) for 30 min alone or in combination with acetate (5 mM) for 4 h in the absence and presence of ACLYi (100 μM) for 16 h or C38 Mab (50 μg) for 6 h. mean ± SEM of triplicates. **(B-C)** Immunoblot analysis of the indicated cancer cell lines stimulated either alone or in combination with α_2_M^*^ (100 pM) for 30 min or acetate (5 mM) for 4 h in the absence and presence of ACLYi (100 μM) for 16 h or C38 Mab (50 μg) for 6 h. **(D-E)** The indicated cancer cell lines stimulated either alone or in combination with α_2_M^*^ (100 pM) for 30 min or acetate (5 mM) for 4 h in the absence and presence of ACLYi (100 μM) for 16 h or C38 Mab (50 μg) for 6 h to quantify the transcript level of ACLY and ACSS1 genes. **(F)** Immunoblot analysis of the indicated cancer cell lines stimulated either alone or in combination with α_2_M^*^ (100 pM) for 30 min or acetate (5 mM) for 4 h in the absence and presence of C38 Mab (50 μg) for 6 h. **(G)** The total cellular concentration of acetyl-CoA was measured in the indicated ACLY-silenced cell lines stimulated with either α_2_M^*^ (100 pM) for 30 min and acetate (5 mM) for 4 h alone or in combination in the absence and presence of C38 Mab (50 μg) for 6 h. mean ± SEM of triplicates. ^*^, *p* values ≤ 0.05. Error bar represent S.D.

To dissect which enzyme is responsible for mediating the α_2_M^*^/CS-GRP78 axis-induced acetyl- CoA production, we treated DU145 and A172 cell lines with C38 Mab and the ACLY inhibitor SB260089 (ACLYi) and then stimulated with α_2_M^*^ in the absence or presence of acetate. In cancer cells ACLYi alone suppressed α_2_M^*^-induced acetyl-CoA production whereas acetate restored this event which indicates that ACSS1 can compensate depending on acetate availability. Importantly, combination of ACLYi with C38 Mab drastically suppressed α_2_M^*^- and acetate-induced acetyl-CoA (Figure 3A) indicating that α_2_M^*^/CS-GRP78 signaling is required for ACLY mediated acetyl-CoA production. These data prompted us to investigate whether ACLY-dependent production of acetyl-CoA potentiates acetylation of histones. We treated multiple cancer cell lines with C38 Mab and ACLYi and then stimulated with α_2_M^*^ in the absence or presence of acetate. We found that ACLYi suppressed α_2_M^*^-induced H2A, H2B, H3 and H4 acetylation whereas acetate rescued this event. C38 Mab effectively inhibited this response (Figure 3B). Thus, combining ACLYi with C38-Mab might effectively suppress histone acetylation. To evaluate the mechanism by which ACLYi and C38 Mab suppresses the histone acetylation we treated the multiple cancer cell lines with C38 Mab and ACLYi and then stimulated with α_2_M^*^ in the absence or presence of acetate. Under this condition ACLYi alone did not inhibit α_2_M^*^- and acetate-induced phosphorylation of AKT (S473) and ACLY whereas combination with C38 Mab effectively suppressed this effect. We also found that ACLYi alone did not affect α_2_M^*^- and acetate-induced ACLY and ACSS1 expression both at the protein and transcript level (Figure 3C, and 3E) but expression was suppressed in combination with C38 Mab. These data indicate that ACLYi only targets events downstream of ACLY-mediated acetyl-CoA production and global histone acetylation but does not have an effect on upstream AKT-mediated ACLY expression. To further substantiate the role of α_2_M^*^/CS-GRP78 signaling in ACLY expression, we silenced ACLY gene in DU145 and A172 cells. The efficiency of ACLY silencing is shown in Figure 3F and Supplementary Figure 4A. siACLY cells were treated with C38 Mab and then stimulated with α_2_M^*^ in the absence or presence of acetate. α_2_M^*^ and acetate did not induce ACLY expression and its phosphorylation whereas it induce ACSS1 expression both at the protein and transcript level and also phosphorylation of AKT (S473) ( Figure 3F and Supplementary Figure 4A). In siACLY cells acetyl-CoA production was decreased whereas α_2_M^*^ and acetate restored its induction, further C38 Mab effectively inhibited this response confirming our ACLY inhibitor results (Figure 3G). We also found that α_2_M^*^ and acetate restored H2A, H2B, H3 and H4 acetylation in siACLY cells whereas C38 Mab effectively inhibited this response (Supplementary Figure 4B). Altogether these data suggest that ACLYi and siACLY cells did not affect ACSS1 expression which indicates that ACSS1-dependent acetylation can compensate for the loss of ACLY function when its substrate is available.

### α_2_M^*^/CS-GRP78 signaling regulates ACLY and ACSS1 expression to induce acetylation of histones and proteins

**Figure 4:**
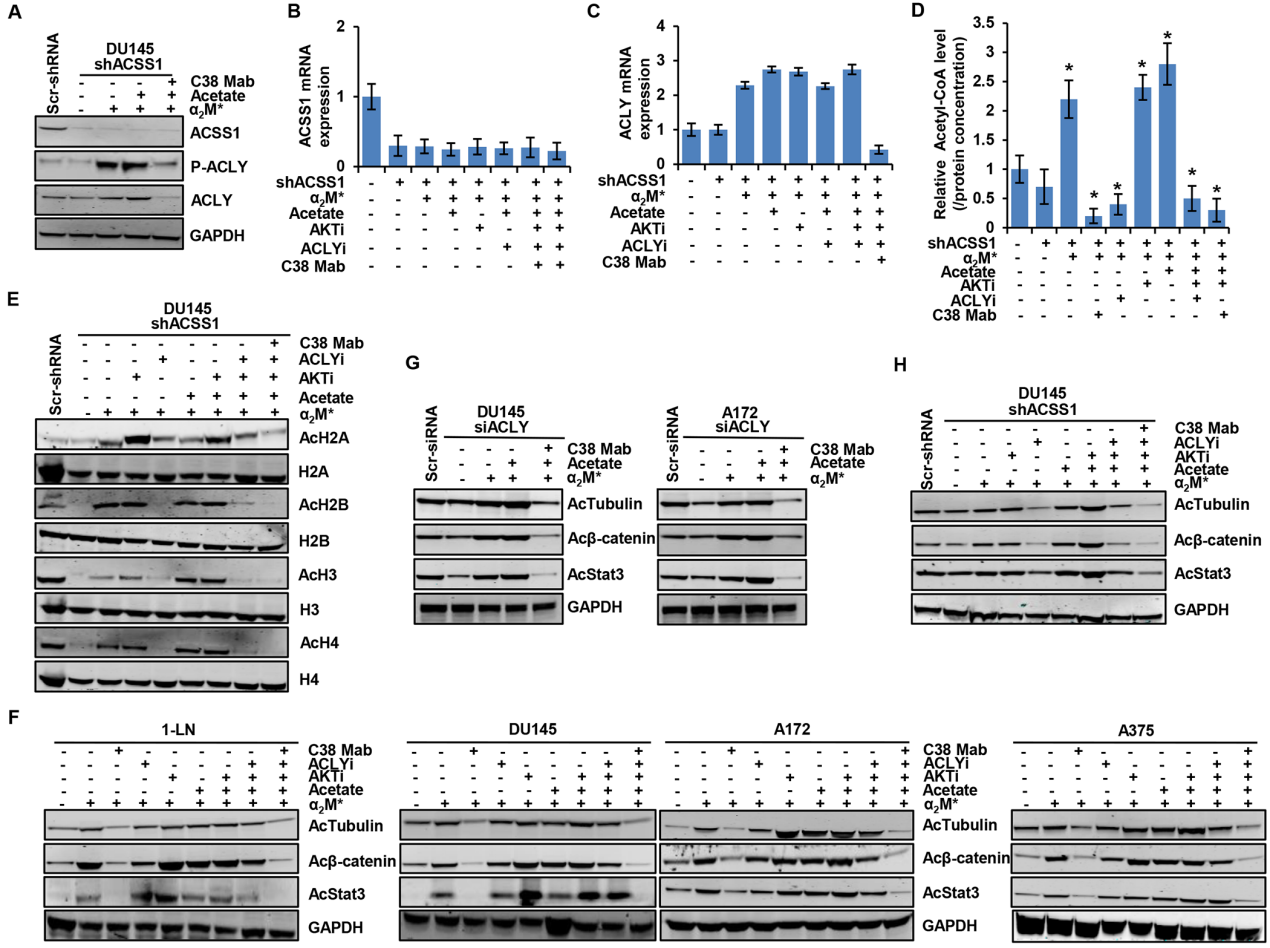
Both ACLY and ACSS1 are involved in α_2_M^*^/CS-GRP78-induced acetylation of proteins **(A)** Immunoblot analysis of indicated proteins in DU145-shACSS1 cells treated with C38 Mab (50 μg) for 6 h and then stimulated with α_2_M^*^ (100 pM) for 30 min and acetate (5 mM) for 4 h. **(B-C)** ACSS1 silencing of DU145 cells were stimulated either alone or in combination with α_2_M^*^ (100 pM) for 30 min or acetate (5 mM) for 4 h in the absence and presence of ACLYi (100 μM) for 16 h, AKTi (GSK690693, 5 μM/L) for 16 h or C38 Mab (50 μg) for 6 h to quantify the transcript level of ACSS1 and ACLY genes. **(D)** The total cellular concentration of acetyl-CoA was measured from ACSS1 silencing of DU145 cells stimulated either alone or in combination with α_2_M^*^ (100 pM) for 30 min or acetate (5 mM) for 4 h in the absence and presence of ACLYi (100 μM) for 16 h, AKTi (GSK690693, 5 μM/L) for 16 h or C38 Mab (50 μg) for 6 h. mean ± SEM of triplicates. **(E)** Immunoblot analysis of histone acetylation levels in ACSS1 silencing of DU145 cells were stimulated either alone or in combination with α_2_M^*^ (100 pM) for 30 min or acetate (5 mM) for 4 h in the absence and presence of ACLYi (100 μM) for 16 h, AKTi (GSK690693, 5 μM/L) for 16 h or C38 Mab (50 μg) for 6 h. **(F)** Immunoblot analysis of protein acetylation in the indicated cell lines stimulated either alone or in combination with α_2_M^*^ (100 pM) for 30 min or acetate (5 mM) for 4 h in the absence and presence of ACLYi (100 μM) for 16 h, AKTi (GSK690693, 5 μM/L) for 16 h or C38 Mab (50 μg) for 6 h. **(G)** Immunoblot analysis of protein acetylation levels in ACLY silencing of indicated cells were treated with C38 Mab (50 μg) for 6 h and then stimulated with α_2_M^*^ (100 pM) for 30 min or acetate (5mM) for 4 h either alone or in combination. **(H)** Immunoblot analysis of protein acetylation levels in ACSS1 silencing of DU145 cells were stimulated either alone or in combination with α_2_M^*^ (100 pM) for 30 min or acetate (5mM) for 4 h in the absence and presence of ACLYi (100 μM) for 16 h, AKTi (GSK690693, 5 μM/L) for 16 h or C38 Mab (50 μg) for 6 h. ^*^, *p* values ≤ 0.05. Error bar represent S.D.

To further investigate the role of α_2_M^*^/CS-GRP78 signaling in regulating either ACLY and ACSS1 expression to induce histone acetylation, we suppressed ACSS1 expression in DU145 cells. The efficiency of ACSS1 silencing is shown in Figure 4A. α_2_M^*^ and acetate did not induce ACSS1 expression whereas it induce ACLY expression and its phosphorylation in these ACSS1-silenced cells. Targeting CS-GRP78 with C38 Mab suppressed α_2_M^*^- and acetate-induced phosphorylation of ACLY and its expression in shACSS1 cells (Figure 4A). To examine the mechanism by which the α_2_M^*^/CS-GRP78 axis regulates ACLY expression in shACSS1 cells we treated with C38 Mab, ACLYi, and AKTi and then stimulated with α_2_M^*^ in the absence or presence of acetate. We found that ACLYi did not affect α_2_M^*^, acetate, and AKTi-induced transcript whereas C38 Mab suppressed this event (Figure 4B, 4C). These data suggest that α_2_M^*^/CS-GRP78-mediated ACLY expression might compensate in this model. To dissect which enzyme is responsible for mediating α_2_M^*^/CS-GRP78-induced acetyl-CoA production, we treated shACSS1 cells with C38 Mab, ACLYi and AKTi and then stimulated with α_2_M^*^ in the absence or presence of acetate. In shACC1 cells acetyl-CoA production were reduced, whereas α_2_M^*^, acetate, and AKTi restored the event. In contrast, ACLYi drastically reduced α_2_M^*^- and AKTi-induced acetyl-CoA production and acetate does not restore this event in this shACSS1 cells. However, we observed that C38 Mab significantly reduced α_2_M^*^, acetate, and AKTi-induced acetyl-CoA in shACSS1 cells (Figure 4D). Thus these data indicate that in the absence of ACSS1 expression, α_2_M^*^/CS-GRP78 signaling permits acetyl-CoA production by regulating ACLY.

We then evaluated histone acetylation in shACSS1 cells by treating with C38 Mab, ACLYi or AKTi alone or in combination and then stimulating with α_2_M^*^ in the absence or presence of acetate. We observed that basal acetylation levels of H2A, H2B, H3 and H4 were reduced in shACSS1 cells when compare to scrambled cells. Interestingly, α_2_M^*^, acetate, and AKTi restored acetylation levels of H2A, H2B, H3 and H4. Furthermore, ACLYi and C38 Mab effectively suppressed α_2_M^*^, acetate, and AKTi rescue of H2A, H2B, H3 and H4 acetylation. Surprisingly, ACLYi effectively suppressed global histone acetylation levels in shACSS1 cells (Figure 4E). These data indicate that ACLYi does not affect ACSS1-mediated histone acetylation and is more effective in an ACLY-dependent manner. Moreover, C38 Mab drastically reduced both ACLY and ACSS1-mediated histone acetylation, suggesting that the α_2_M^*^/CS-GRP78 signaling pathway might regulate both the enzymes in an AKT-dependent manner to regulate histone acetylation.

We next sought to determine the mechanism by which α_2_M^*^/CS-GRP78 signaling regulates ACLY-induced histone acetylation in the shACSS1 cells. We treated the cells as in Figure 4E. C38 Mab effectively blocked α_2_M^*^-, acetate- and AKTi-induced phosphorylation of AKT (S473) and ACLY and it also suppressed ACLY expression. In contrast, ACLYi did not have any effect on the phosphorylation of AKT (S473) and ACLY (Supplementary Figure 4C). These data indicate that in the absence of ACSS1 expression, acetate induces ACLY expression by activating AKT to promote acetyl-CoA production and histone acetylation. Collectively, these data support our hypothesis that the α_2_M^*^/CS-GRP78 signaling axis regulates both ACLY and ACSS1 depends on nutrient availability for the acetyl-CoA production and histone acetylation.

To determine whether α_2_M^*^/CS-GRP78 signaling-dependent acetyl-CoA production selectively affects acetylation of histones or has equivalent effects on the acetylation of other cellular proteins. We stimulated multiple cancer cell lines with α_2_M^*^ and acetate alone or in combination in the absence and presence of AKTi, ACLYi, and C38 Mab and probed for the indicated proteins. Targeting CS-GRP78 with C38 Mab drastically suppressed α_2_M^*^-, acetate- and AKTi-induced acetylation of tubulin, β-catenin, and Stat3 whereas ACLYi had a moderate effect on these acetylated proteins. Combination of C38 Mab with ACLYi or alone had a more pronounced effect on suppression of protein acetylation when compared to ACLYi alone (Figure 4F). We next investigated whether ACLY or ACSS1 is required for the acetylation of proteins. We treated the siACLY cells with C38 Mab and then stimulated with α_2_M^*^ with or without acetate. ACLY-silenced cells slightly decreased the acetylation of proteins whereas α_2_M^*^ and acetate restored protein acetylation. Importantly, C38 Mab drastically suppressed α_2_M^*^- and acetate- restored acetylation of tubulin, β-catenin and Stat3 (Figure 4G). These data indicate in the absence of ACLY expression α_2_M^*^/CS-GRP78 signaling regulates ACSS1 expression to induce protein acetylation. To confirm this hypothesis we treated the shACSS1 cells as in Figure 4F. Silencing of ACSS1 slightly decreased acetylation of tubulin, β-catenin, and Stat3 whereas α_2_M^*^, acetate, and AKTi alone or in combination restores protein acetylation (Figure 4H). In contrast to the scrambled cells (Figure 4F), ACLYi more effectively suppressed protein acetylation in the shACSS1 cells which indicates it is more specific to ACLY activity and does not affect ACSS1-induced acetylation. Moreover, C38 Mab drastically suppressed α_2_M^*^-, acetate- and AKTi-induced acetylation of tubulin, β-catenin and Stat3 (Figure 4H). Thus α_2_M^*^/CS-GRP78 signaling is required for both ACLY- and ACSS1-mediated acetylation of proteins.

### α_2_M^*^/CS-GRP78 signaling restores acetyl-CoA production and histone acetylation under hypoxia

**Figure 5:**
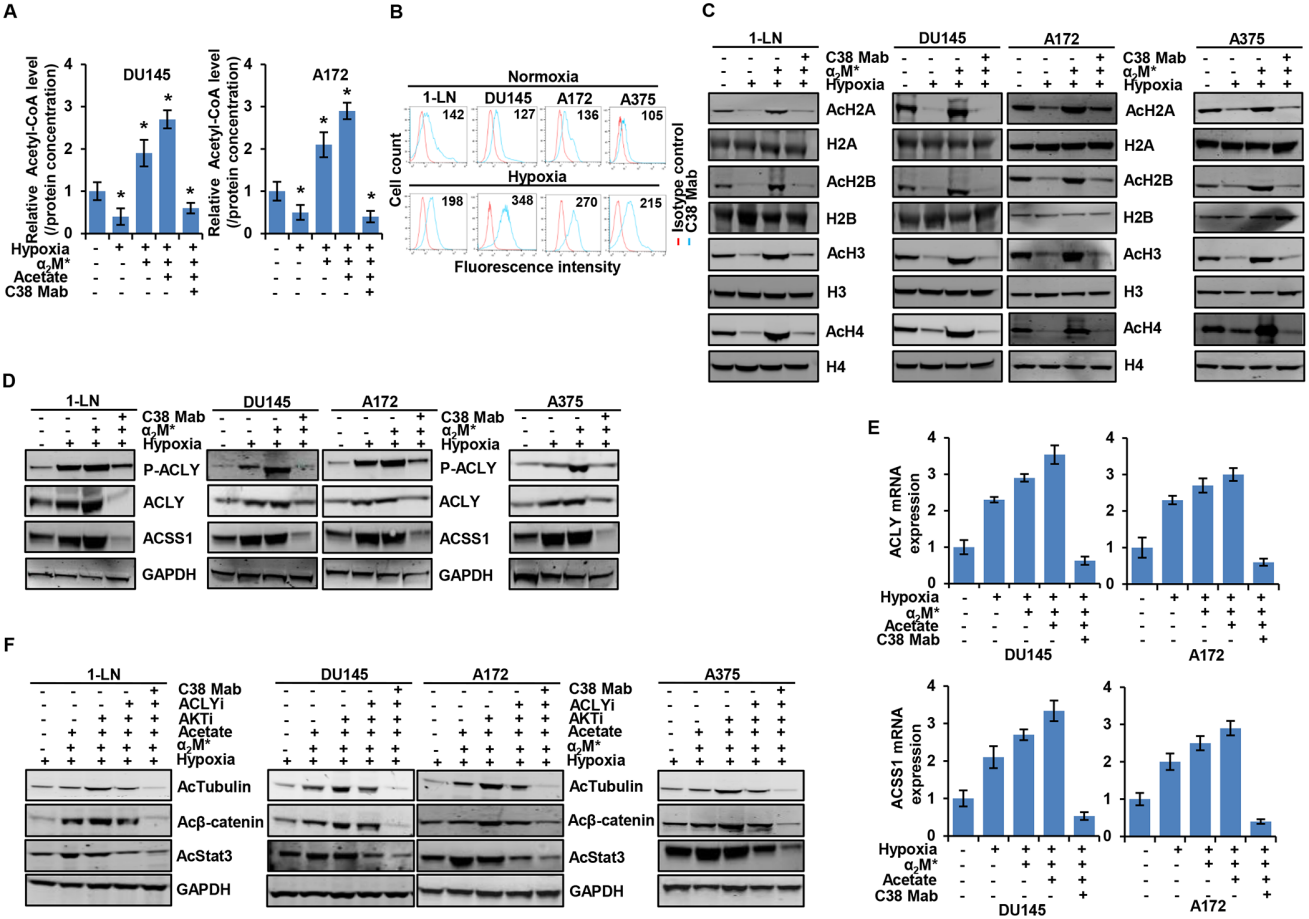
α_2_M^*^/CS-GRP78 rescues hypoxia-induced reduction of histone acetylation **(A)** The total cellular concentration of acetyl-CoA was measured in the indicated cell lines under normoxia and hypoxia conditions. The cells were treated with C38 Mab (50 μg) for 6 h and then stimulated with α_2_M^*^ (100 pM) for 30 min and acetate (5 mM) for 4 h. mean ± SEM of triplicates. **(B)** Surface expression of GRP78 was detected in the indicated cancer cell lines under normoxia or hypoxia by flow cytometric analysis of nonpermeabilized cells. Surface GRP78 was visualized with murine monoclonal antibody C38, followed by fluorescently labeled secondary antibody and the relative to matched isotype control. Positively stained cells are represented as the area under the respective histogram, and mean fluorescence intensity (MFI) values are shown. **(C-D)** Immunoblot analysis of the indicated cancer cell lines under normoxia or hypoxia treated with C38 Mab (50 μg) for 6 h and then stimulated with α_2_M^*^ (100 pM) for 30 min. **(E)** Indicated cancer cell lines under normoxia or hypoxia were treated with C38 Mab (50 μg) for 6 h and then stimulated with α_2_M^*^ (100 pM) for 30 min and acetate (5 mM) for 4 h to quantify the transcript level of ACLY and ACSS1 genes. **(F)** Immunoblot analysis of protein acetylation levels in indicated cancer cell lines under normoxia or hypoxia stimulated either alone or in combination with α_2_M^*^ (100 pM) for 30 min or acetate (5 mM) for 4 h in the absence and presence of ACLYi (100 μM) for 16 h, AKTi (GSK690693, 5 μM/L) for 16 h or C38 Mab (50 μg) for 6 h. ^*^, *p* values ≤ 0.05. Error bar represent S.D.

Hypoxic stress is a critical player in tumorigenesis and tumor development [44]. Cancer cells demand distinctive extracellular nutrients and reprogram the metabolic pathways to survive and proliferate under hypoxic condition [45]. To delineate the changes in the acetyl-CoA production of normoxic versus hypoxic cancer cells, we measured acetyl-CoA in DU145 and A172 cell lines treated with C38 Mab under hypoxia and then stimulated with α_2_M^*^ in the absence or presence of acetate. We found that acetyl-CoA production was decreased under hypoxic conditions. Moreover, C38 Mab suppressed the α_2_M^*^ and acetate restored acetyl-CoA production, indicating the critical role of CS-GRP78 under hypoxia (Figure 5A). We then screened CS-GRP78 in multiple cancer cell lines under either normoxia (Figure 5B Upper panel) or hypoxia (Figure 5B lower panel). We noted a significant increase of CS-GRP78 in hypoxia compared to normoxia. This indicates that under hypoxic conditions, CS-GRP78 signaling might modulate acetyl-CoA production.

To characterize the role of α_2_M^*^/CS-GRP78 signaling in epigenetic regulation under hypoxia we treated multiple cancer cell lines with C38 Mab under hypoxia and then stimulated with α_2_M^*^ and acetate. We found that C38 Mab suppressed the α_2_M^*^ and acetate restored H2A, H2B, H3 and H4 acetylation during hypoxia in different cancer cell lines (Figure 5C and Supplementary Figure 5A). These data imply that α_2_M^*^/CS-GRP78 signaling is implicated in the regulation of histone acetylation under hypoxia. To investigate the mechanism by which the α_2_M^*^/CS-GRP78 axis regulates acetyl-CoA production under hypoxia we treated different cancer cell lines with C38 Mab under hypoxia and then stimulated with α_2_M^*^ and acetate. Interestingly, phosphorylation of ACLY and induction of ACLY and ACSS1 expression at both the protein and mRNA levels were increased under hypoxia compared with normoxia (Figure 5D, 5E and Supplementary Figure 5B) which was consistent with other studies [13, 46] and discrepant from Gao et al [38]. This may be due to experimental conditions, such as different cell lines used. Moreover, stimulation with α_2_M^*^ and acetate increased phosphorylation of ACLY and upregulated both the protein and mRNA expression of ACLY and ACSS1 under hypoxia whereas C38 Mab suppressed these events (Figure 5D, 5E and Supplementary Figure 5B). These data indicate that α_2_M^*^/CS-GRP78 axis plays an important role in regulating ACLY and ACSS1 expression for histone acetylation in cancer cells adapting hypoxia.

In hypoxic cancer cells, we found increased phosphorylation of AKT (S473) while α_2_M^*^ and acetate augmenting this event. Moreover C38 Mab suppressed hypoxia-induced, α_2_M^*^ and acetate augmented phosphorylation of AKT (S473) (Supplementary Figure 5C). These data confirm that α_2_M^*^/CS-GRP78 signaling augments the AKT pathway in hypoxic cancer cells. We further investigated whether α_2_M^*^/CS-GRP78 signaling-induced histone acetylation during hypoxia was associated with acetylation of proteins. We treated the various hypoxic cancer cell lines with AKTi, ACLYi, and C38 Mab and then stimulated with α_2_M^*^ and acetate. We found that C38 Mab drastically suppressed α_2_M^*^, acetate and AKTi rescued acetylation of tubulin, β-catenin and Stat3, under hypoxia. Moreover, ACLYi moderately reduced the rescue of acetylated proteins by α_2_M^*^ and acetate whereas combination of ACLYi with C38 Mab effectively suppressed this events (Figure 5F and Supplementary Figure 5D). These data suggest that ACSS1 can compensate in this model, depending on substrate availability. Collectively, these data imply that under hypoxic α_2_M^*^/CS-GRP78 signaling regulates the acetylation of proteins in an AKT-dependent manner.

To dissect which enzyme is responsible for mediating the α_2_M^*^/CS-GRP78-mediated acetylation of proteins we treated shACSS1 cells under hypoxia as in Figure 5F. To our surprise α_2_M^*^, acetate, and AKTi were capable of rescuing acetylation of tubulin, β-catenin and Stat3 in shACSS1 cells whereas C38 Mab effectively suppressed the rescue of protein acetylation. Moreover ACLYi suppressed the rescued acetylated proteins in shACSS1 which indicates ACLYi is more potent in the absence of ACSS1 expression (Supplementary Figure 5E). These observations suggest that the α_2_M^*^/CS-GRP78 axis regulates ACLY mediated histone acetylation in the absence of ACSS1 expression under hypoxia. Collectively, these data support our notion that α_2_M^*^/CS-GRP78 signaling is required for both the ACLY and ACSS1 mediated acetylation of proteins.

### α_2_M^*^/CS-GRP78 signaling is required for the glucose consumption and lactate production during acetylation of histone

**Figure 6:**
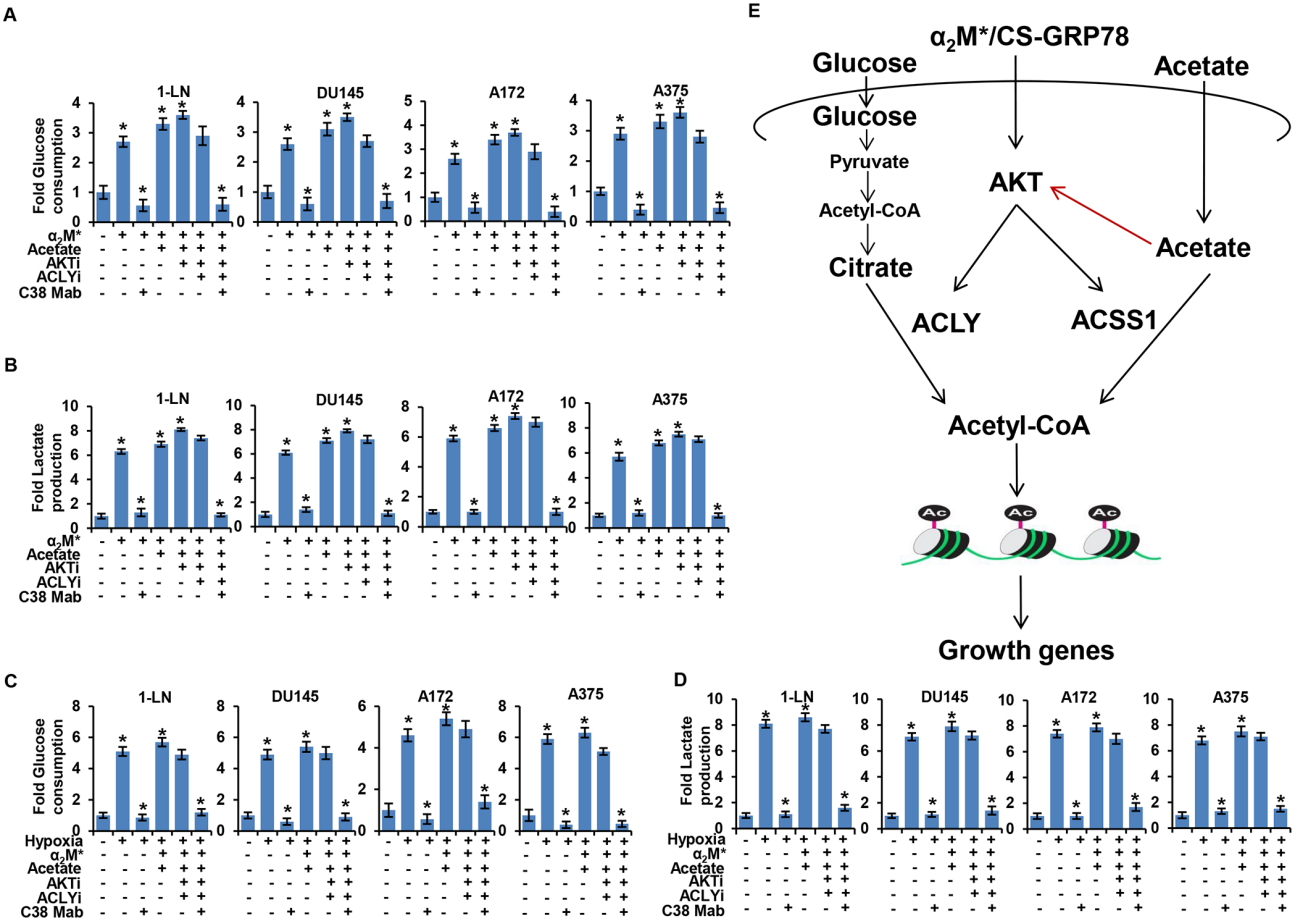
α_2_M^*^/CS-GRP78 signaling dictates aerobic glycolysis in an AKT dependent manner **(A-B)** Glucose consumption and lactate production were measured in indicated cancer cell lines stimulated either alone or in combination with α_2_M^*^ (100 pM) for 30 min or acetate (5 mM) for 4 h in the absence and presence of ACLYi (100 μM) for 16 h, AKTi (GSK690693, 5 μM) for 16 h or C38 Mab (50 μg) for 6 h. mean ± SEM of triplicates. **(C-D)** Glucose consumption and lactate production were measured in the indicated cancer cell lines under normoxia or hypoxia were stimulated either alone or in combination with α_2_M^*^ (100 pM) for 30 min or acetate (5 mM) for 4 h in the absence and presence of ACLYi (100 μM) for 16 h, AKTi (GSK690693, 5 μM/L) for 16 h or C38 Mab (50 μg) for 6 h. mean ± SEM of triplicates. **(E)** A model for α_2_M^*^/CS-GRP78 axis-dependent induction of histone acetylation. In addition to its ability to promote AKT pathway α_2_M^*^/CS-GRP78 axis also functions as a regulator of epigenetic metabolites to induce histone acetylation and tumor growth. Red arrow indicates that acetate regulates the AKT pathway through a feedback loop mechanism in a CS-GRP78 dependent manner. ^*^, *p* values ≤ 0.05.

To investigate the biological effect on cancer metabolism induced by α_2_M^*^/CS-GRP78-mediated histone acetylation we examined glucose consumption and lactate production in various cancer cell lines stimulated with α_2_M^*^ and acetate alone or in combination in the absence and presence of AKTi, ACLYi, and C38 Mab. We found that C38 Mab significantly reduced α_2_M^*^, acetate and AKTi induced glucose uptake and lactate consumption whereas ACLYi had a moderate effect. However, combination of C38 Mab with ACLYi effectively reduced glucose uptake and lactate consumption (Figure 6A, 6B). To observe the effect of ACLY and ACSS1 on glucose and lactate metabolism we treated the shACSS1 cells as in Figure 6A, 6B. C38 Mab and ACLYi significantly reduced α_2_M^*^, acetate and AKTi-induced glucose uptake and lactate consumption in shACSS1 cells (Supplementary Figure 5A). These results demonstrate that α_2_M^*^/CS-GRP78 signaling regulates glucose and lactate metabolism during acetyl-CoA production.

We next sought to determine whether α_2_M^*^/CS-GRP78 signaling regulates glucose and lactate metabolism under hypoxia. We treated the various cancer cell lines with AKTi, ACLYi and C38 Mab alone or in combination during hypoxia and then stimulated with α_2_M^*^ and acetate. We observed that C38 Mab significantly reduced α_2_M^*^, acetate and AKTi induced glucose uptake and lactose production whereas ACLYi caused a moderate reduction. Moreover, combination of C38 Mab with ACLYi significantly reduced glucose uptake and lactose production (Figure 6C, 6D). When we employed another non-ATP-competitive AKT inhibitor (AKTi-1/2) it blocked hypoxia mediated glucose uptake and lactose production. We also observed that under hypoxic condition AKTi-1/2 significantly reduced α_2_M^*^ and acetate induced glucose uptake and lactose production (Supplementary Figure 6B). These data imply that α_2_M^*^/CS-GRP78 signaling regulates glucose and lactate metabolism in an AKT-dependent manner under hypoxia.

## DISCUSSION

Dysregulation of cellular metabolism is a hallmark of cancer [1, 2]. It is important to decipher the mechanism(s) by which cancer cells use this metabolic shift to maximize their growth. In the present study we report that the α_2_M^*^/CS-GRP78 signaling axis regulates ACLY and ACSS1 expression by promoting AKT activation which ensures continuous acetyl-CoA production and global histone acetylation even during nutrient limitation.

A recent report demonstrates that glucose availability affects histone acetylation in response to growth factor stimulation, linking growth factor-induced increases in nutrient metabolism to the regulation of histone acetylation and gene expression [22, 47]. Further, cancer cells avidly take up and use acetate as a fuel source and for macromolecular biosynthesis through its conversion into acetyl-CoA [7, 9, 12, 38]. Our initial studies demonstrated that the α_2_M^*^/CS-GRP78 axis regulates fatty acid synthesis and acetate uptake in prostate cancer cells [36]. At present, the nutrient switch from glucose to acetate and its role in cellular regulation and cancer are less well understood. Here, we demonstrate that α_2_M^*^/CS-GRP78 signaling regulates cancer cell by utilizing glucose and acetate as a carbon source for the production of acetyl-CoA. Moreover, acetyl-CoA production shows a similar response in α_2_M^*^ stimulation alone without acetate addition yet each remains responsive to CS-GRP78. These results suggest that CS-GRP78 regulates acetyl-CoA production in response to environmental constraints for tumor proliferation. Our findings highlight the complex mechanism in cancer cells in that the α_2_M^*^/CS-GRP78 axis regulates both acetate and glucose dependent acetyl-CoA production which might be an adaptive mechanism in rapidly proliferating tumors.

The major acetyl group donor for protein and histone acetylation is acetyl-CoA, which is a key metabolic intermediate produced and consumed my many metabolic reactions [16, 25]. Acetyl-CoA promotes growth and proliferation in yeast, through histone acetylation at genes involved in these processes [21, 48]. Moreover, acetyl-CoA and acetylation regulate many metabolic enzymes [49] and a role for acetyl-CoA in suppressing autophagy has also been recently reported [50, 51]. Therefore, elucidating the mechanisms that control tumor histone acetylation levels are clinically important. We show here that α_2_M^*^/CS-GRP78 signaling is a key determinant for global histone acetylation. We extended the previous finding [22] and provide evidence that α_2_M^*^/CS-GRP78 axis function as a upstream regulator of AKT dependent metabolic reprogramming to promote high acetyl-CoA production. Besides functioning as a pro-proliferative signaling pathway, the α_2_M^*^/CS-GRP78/AKT axis regulates glucose and acetate-derived acetyl-CoA production to induce histone acetylation levels and function as an epigenetic metabolite to promote cancer cell survival and proliferation.

We recently showed that the α_2_M^*^/CS-GRP78 axis regulates ACLY-dependent lipogenesis through the AKT pathway for the proliferation of glycolytically converted tumor cells [36]. Here we showed further that the α_2_M^*^/CS-GRP78 signaling axis-mediated AKT activation ensures continuous acetyl-CoA production even during nutrient limitation by promoting the phosphorylation and activation of ACLY. A striking finding of this study is that acetate regulates the expression of ACLY and activating by its phosphorylation. This observation highlights its possible that acetate might regulate ACLY through feedback loop mechanism to support fatty acid and lipid biosynthesis during nutrient limitation. Future studies are warranted to confirm this prediction. Our work also suggests that α_2_M^*^/CS-GRP78 signaling modulates the rate of acetyl-CoA generation by salvage pathways independent of ACLY which can be sufficient to maintain macromolecular synthesis that supports tumor growth. As discussed above, the ACLY-dependent rise in acetyl-CoA production is necessary but not sufficient for increase in histone acetylation.

It is also know that acetate generates acetyl-CoA through ACSS1 under conditions of metabolic stress [47]. Interestingly, our data show that the α_2_M^*^/CS-GRP78 axis regulates both ACLY-mediated glucose and ACSS1-mediated acetate contribution to acetyl-CoA production and histone acetylation in an AKT-dependent manner. These results suggest that cancer cells utilize this oncogenic pathway for both epigenetic modifications and as a carbon source to meet growth needs. A surprising implication of this study is that acetate regulates the expression of ACLY and ACSS1 through a feed forward activation of AKT signaling to maintain acetyl-CoA production which drives histone acetylation and tumor proliferation. Further study is needed to understand how this feedback loop regulates global histone acetylation to impact tumor proliferation and progression. These observations confirm the previous findings that tumor cells maintain the ability to acetylate Rictor in response to acetate when glucose is not available and demonstrate the complexity of nutrient-dependent maintenance of mTORC2 signaling [41]. It also confirms the previous prediction that cancer cells uses nutrients to escape targeted therapies and provides rationale that combined inhibition of PI 3-kinase/EGFR pathways and metabolic pathways is essential. In glioblastoma cancer cells, glucose and acetate-dependent maintenance of mTORC signaling may contribute to temozolomide resistance [52]. Moreover, ACLY inhibitors are ineffective for all cancers [53]. However, our data predict that targeting the α_2_M^*^/CS-GRP78 signaling provides a potential advantage over cancer with metabolic reprogramming because CS-GRP78 is a potent activator of PI 3-kinase/AKT pathway which regulates both glucose and lipid metabolism [33, 36, 54] as well as ACLY and ACSS1 expression levels and their activity. Therefore targeting the α_2_M^*^/CS-GRP78 axis with C38 Mab might be effective therapeutic strategy by abrogating the metabolic effects downstream of PI 3-kinase/AKT activation.

Alterations in histone modification also plays important roles in acetylated of other proteins in cancer cells [21]. Acetylated β-catenin, tubulin, and stat3 are altered in various cancer cells [55–57]. Here we show that the α_2_M^*^/CS-GRP78 axis-induced ACLY and ACSS1 expression triggers acetyl-CoA production which increased the acetylated of these proteins. As protein acetylation is linked to metabolism in several previous studies [3, 47, 49, 58], we predict that the α_2_M^*^/CS-GRP78 signaling axis might triggers protein acetylation with respect to intracellular acetyl-CoA levels. They may represent a widely conserved mechanism by which cell proliferation is coordinated with metabolic state.

Micro environmental conditions, such as hypoxia, also potently reprogram cellular metabolism [13, 38]. We anticipate that α_2_M^*^/CS-GRP78 signaling plays a crucial role in integrating cellular metabolism with signal transduction to drive tumor growth under hypoxic condition. Our study revealed a mechanistic solution for the need of α_2_M^*^/CS-GRP78 axis and acetate in cancer cells by demonstrating that under tumor like tissue culture conditions there is a switch in nutrient utilization from glucose and acetate to support histone acetylation and acetyl-CoA production under hypoxia. In support this mechanism we found that CS-GRP78 was indeed significantly upregulated under hypoxia. Taken together, this study demonstrates that the α_2_M^*^/CS-GRP78 axis regulates acetate to function as an epigenetic metabolite to promote cancer cell survival under hypoxic stress. In this study, we demonstrate that the α_2_M^*^/CS-GRP78 axis functions as an epigenetic by regulating metabolite to enhance acetyl-CoA production and histone acetylation for tumor growth (Figure 6E). First, global histone acetylation sites can be stimulated by α_2_M^*^ in a both time and dose-dependent manners and suggests that α_2_M^*^ function like acetate to promote acetylation. Notably, α_2_M^*^/CS-GRP78 axis derived acetyl-CoA is found to be incorporated not only into histone acetylation but also into acetylated proteins. Second, the α_2_M^*^/CS-GRP78 axis induced ACLY and ACSS1 expression is then associated with acetyl-CoA production which activates acetylation of histone and proteins for tumor growth. Third, the α_2_M^*^/CS-GRP78 mediated AKT activation regulates ACLY and ACSS1 expression which might plays a critical role in integrating cellular metabolism with signal transduction to drive tumor growth in response to environmental constraints. Fourth, acetate itself regulates AKT pathway for the ACLY and ACSS1 expression through feedback loop mechanism. Last and most important targeting α_2_M^*^/CS-GRP78 with C38 Mab abrogates α_2_M^*^ and acetate-induced histone and other protein acetylation necessary for tumor proliferation and survival under hypoxic stress. Collectively, besides its ability to induce fatty acid synthesis, our study reveals a new mechanism of epigenetic regulation by the α_2_M^*^/CS-GRP78 axis to increase histone acetylation and promote cell survival under unfavorable condition.

## MATERIALS AND METHODS

### Cell culture

1-LN prostate cancer cells were the kind gift from Dr. Philip Walther, Department of Surgery, Duke University Medical Center. They now reside in our laboratory and are available on request. DU145 prostate cancer cells, A375 melanoma cells and A172 glioma cells were purchased from the Duke Cell Culture Facility. 1-LN and DU145 cells were maintained in RPMI 1640 medium (Sigma) containing 10% fetal bovine serum (FBS) 1% penicillin/streptomycin at 37°C in a 5% CO_2_-humidified atmosphere. A375 and A172 cells were in DMEM (high glucose, Gibco-life Technologies) containing 10% FBS, 1% penicillin/streptomycin at 37°C in a 5% CO_2_-humidified atmosphere. For all the experimental conditions unless otherwise mentioned the cells were grown in corresponding media with 1% serum.

### Antibodies and reagents

Antibodies recognizing Acetyl-Histone H2A, H2B, H3 and H4, Hisotne H2A, H2B, H3 and H4, P-ACLY (Ser455), ACLY, ACeCS1, P-Akt (Ser473), P-AKT (Thr308), AKT, P-IRS1(Ser612), IRS1, P-FoxO1(Thr24)/FoxO3a(Thr32), P-ERK1/2(Thr202/Tyr204), ERK1/2, P-PRAS40(T246), PRAS40, P-GSK3β(S21), GSK3β, Acetyl-Stat3(Lys685), Acetyl-β-catenin (Lys49), Acetyl-α-Tubulin (Lys40), were purchased from Cell Signaling Technologies. GAPDH antibody was purchased from Genscript. Secondary antibodies conjugated with Alexa fluor 680, Alexa fluor 790, and Alexa fluor 647 were purchased from Invitrogen. IR dye 800 CW was purchased from Rockland. Acetate solution, High sensitivity Glucose assay kit and Lactose assay kit were purchased from Sigma-Aldrich. SB-204990, GSK690693 and AKTi-1/2 were purchased from Selleckchem. α_2_M^*^ was prepared as described previously [59]. GRP78 murine monoclonal antibody (C38) was produced in our laboratory [35].

### Peptides

GRP78 wild type peptide (WT) CLIGRTWNDPSVQQDIKFL (Leu^98^-Leu^115^), and a scrambled peptide GTNKSQDL WIPQLRDVFI were purchased from Genemed Synthesis, Inc.

### Small interfering RNA (siRNA) interference and Lentiviral transfections

siRNAs targeting human ACLY (Dharmacon, #L-004915-00 and non-specific siRNA (Dharmacon #D-001810-01-20) were transfected into DU145 and A172 cells with Lipofectamine 2000 reagent according to the manufactures instructions. shACSS1 lentiviral particles (Clone ID TRCN0000045381) were obtained from Sigma and transfected in to DU145 cells according to manufacturer's instructions. After transfection with shACSS1 vector, DU145 cells were selected with 2 mg/ml puromycin.

### Flow cytometry

CS-GRP78 was analyzed by flow cytometry as described previously [26]. The mean fluorescence intensity (MFI) of the signal was calculated by Flow Jo^®^ software and signal obtained from GRP78 was normalized with that obtained from isotype controls.

### Acetyl-CoA measurement

The intracellular acetyl-CoA level was calculated in a pmol range using the PicoProbe Acetyl-CoA assay kit (Abcam). Fluorescence was measured using Ex/Em=535/589 nm with a plate reader. After correcting the background from all readings, values for each sample were determined and normalized by protein concentration of each sample.

### Glucose and lactate measurements

Tumor cell lines were plated at a concentration of 100,000 cells/ml in the corresponding media. The following day tumor cells were replaced with fresh serum free medium with ACLYi, AKTi (GSK690693), AKTi-1/2 and C38 Mab and then stimulated with α_2_M^*^ and acetate. Medium samples were collected for the assay. Glucose and lactate were measured using colorimetric kits according to the manufacturer's instructions. High sensitivity Glucose assay kit and Lactose assay kit were obtained from Sigma.

### Immunoblotting and immunoprecipitation

Protein extracts, immunoblotting, and immunoprecipitate analysis were performed as described previously [26] and all blots are representative of a minimum two independent experiments.

### Quantitative real-time PCR

Total RNA were prepared from cells using the Quick RNA Mini prep (Zymo Research) and cDNAs were generated using the iScript cDNA synthesis kit (BIO-RAD). SYBR Green reactions were done using a BioRad CFX96 quantitative real-time PCR (qRT-PCR) system. For data analysis, raw counts were normalized to the housekeeping gene averaged for the same time point and condition (ΔC_t_). Counts are reported as fold change relative to the untreated control (2^−ΔΔCt^). All primers were designed by IDT and synthesized by eurofins mwg operon. Primers sequences were as follows: ACLY Forward primer 5’ – CAT CGC AAA CTT CAC CAA CG – 3’; ACLY Reverse primer 5’-AGA TTG TGA CTT CGT GCT CC-3’; ACSS1 Forward primer 5’-TTG AGA GCA CCC CAG TTT ATC-3’; ACSS1 Reverse primer 5’-GCA TCA CCG TAT TTC AGC AAC-3’.

### Hypoxic treatment

For hypoxic treatment, cancer cells were placed with in enclosed chamber from Biospherix at 37°C temperature controlled incubator. The incubator was humidified with a water tray and the oxygen concentration was maintained via regulated infusion of premixed gas (1% O_2_, 94% N_2_ and 5% CO_2_). The oxygen concentration within the chamber was continuously monitored.

### Statistical analysis

Data are presented as mean ± SEM, unless otherwise stated. A student's T test was used to compare two groups for statistical significance analysis. P-Value ≤ 0.05 were considered as significant.

## SUPPLEMENTARY MATERIALS FIGURES



## Abbreviations

α_2_M: α_2-_Macroglobulin
ACLY: ATP-citrate lyase
ACSS1: Acetyl-CoA Synthetase
AC: acetyl
CS-GRP78: Cell Surface GRP78
Scr: scrambled
