# Supplementary material for: Cell surface GRP78 promotes tumor cell histone acetylation through metabolic reprogramming: a mechanism which modulates the Warburg effect
Source: Oncotarget. 2017 Nov 14;8(64):107947–63. doi: 10.18632/oncotarget.22431 (PMC5746117; doi:10.18632/oncotarget.22431)
Supplement: Supplementary file 1 [file oncotarget-08-107947-s001.pdf]

Cell surface GRP78 promotes tumor cell histone acetylation through metabolic reprogramming: a mechanism which modulates the Warburg effect

SUPPLEMENTARY MATERIALS

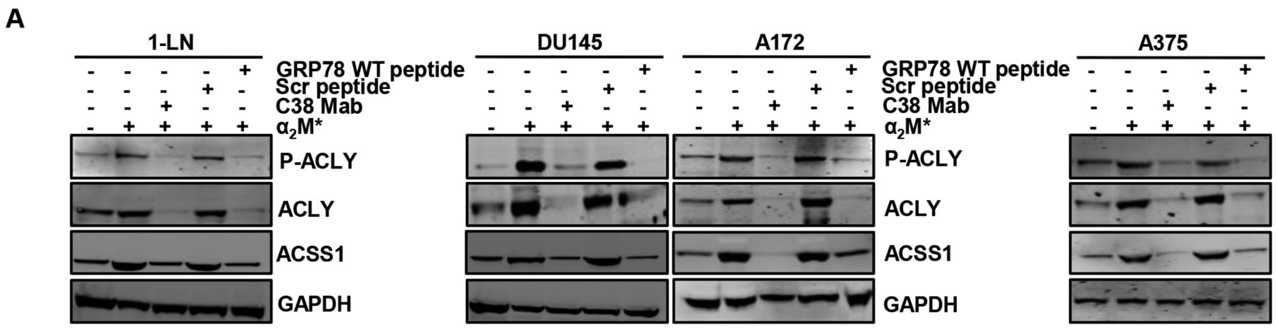

Supplementary Figure 1: α_2M^* signals through CS-GRP78 to control protein levels of ACLY and ACSS1. **(A)** Various cancer cell lines were stimulated with α_2M^* (100 pM) for 30 min in the absence or presence of Scr peptide (100 pM for 1h), or GRP78 peptide (100 pM for 1h) or C38 Mab (50 µg) for 6 h and then immunoblotted for indicated proteins.

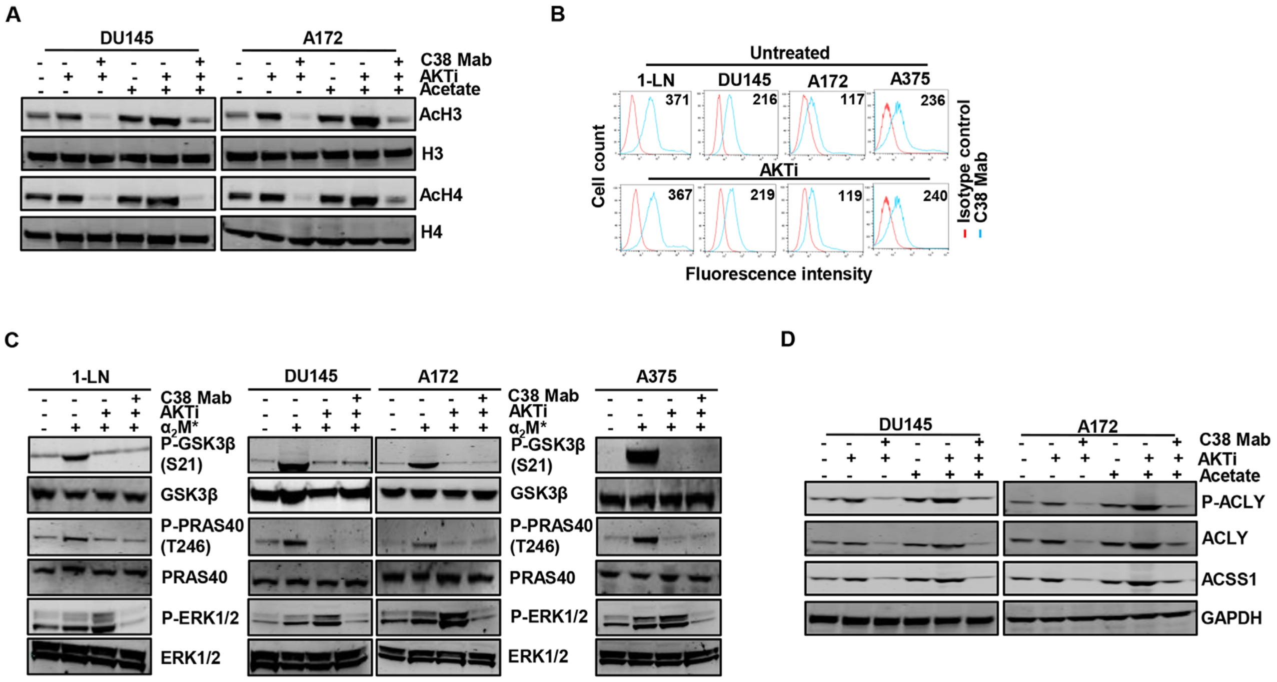

Supplementary Figure 2: α_2M^*/CS-GRP78 signaling is critical for AKT feedback mechanism. **(A)** Immunoblot analysis of the indicated cancer cell lines stimulated with acetate (5 mM) for 4h in the absence and presence of AKTi (GSK690693, 5 µM/L) for 16 h or C38 Mab (50 µg) for 6 h. **(B)** Surface expression of GRP78 was detected by flow cytometric analysis of nonpermeabilized cells in the indicated cancer cell lines treated with AKTi (GSK690693, 5 µM/L) for 16 h. Surface GRP78 was visualized with murine monoclonal antibody C38, followed by fluorescently labeled secondary antibody and the relative to matched isotype control. Positively stained cells are represented as the area under the respective histogram, and mean fluorescence intensity (MFI) values are shown. **(C)** Immunoblot analysis of AKT pathway in indicated cancer cell lines stimulated with α_2M^* (100 pM) for 30 min in the absence and presence of AKTi (GSK690693, 5 µM/L) for 16 h or C38 Mab (50 µg) for 6 h. **(D)** Immunoblot analysis of the indicated cancer cell lines stimulated with acetate (5 mM) for 4h in the absence and presence of AKTi (GSK690693, 5 µM/L) for 16 h or C38 Mab (50 µg) for 6 h.

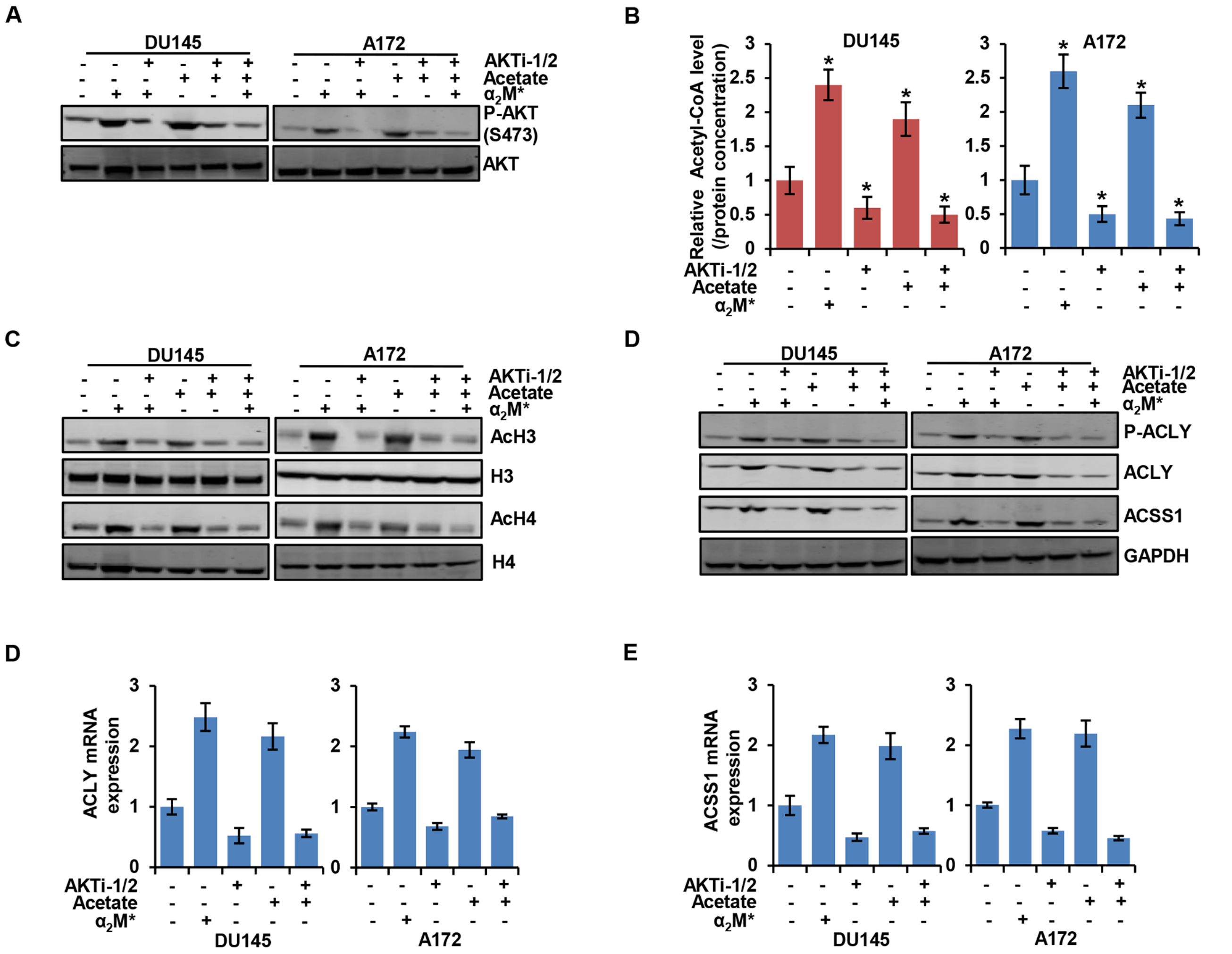

Supplementary Figure 3: α_2M^*/CS-GRP78 regulates histone acetylation in a AKT dependent manner. (A) Immunoblot analysis of the indicated cancer cell lines stimulated either alone or in combination with α_2M^* (100 pM) for 30 min or acetate (5 mM) for 4h in the absence and presence of AKTi-1/2 (10 µM) for 16 h. **(B)** Total cellular concentration of acetyl-CoA was measured in indicated cell lines treated with AKTi-1/2 (10 µM) for 16 h and then stimulated with α_2M^* (100 pM) for 30 min and acetate (5mM) for 4h alone. mean ± SEM of triplicates. **(C-D)** Immunoblot analysis of the indicated cancer cell lines stimulated either alone or in combination with α_2M^* (100 pM) for 30 min or acetate (5 mM) for 4h in the absence and presence of AKTi-1/2 (10 µM) for 16 h. **(E-F)** Indicated cancer cell lines were treated with AKTi-1/2 (10 µM) for 16 h and then stimulated with α_2M^* (100 pM) for 30 min and acetate (5 mM) for 4h alone to quantify the transcript level of ACLY and ACSS1 genes. *, p values ≤ 0.05. Error bar represent S.D.

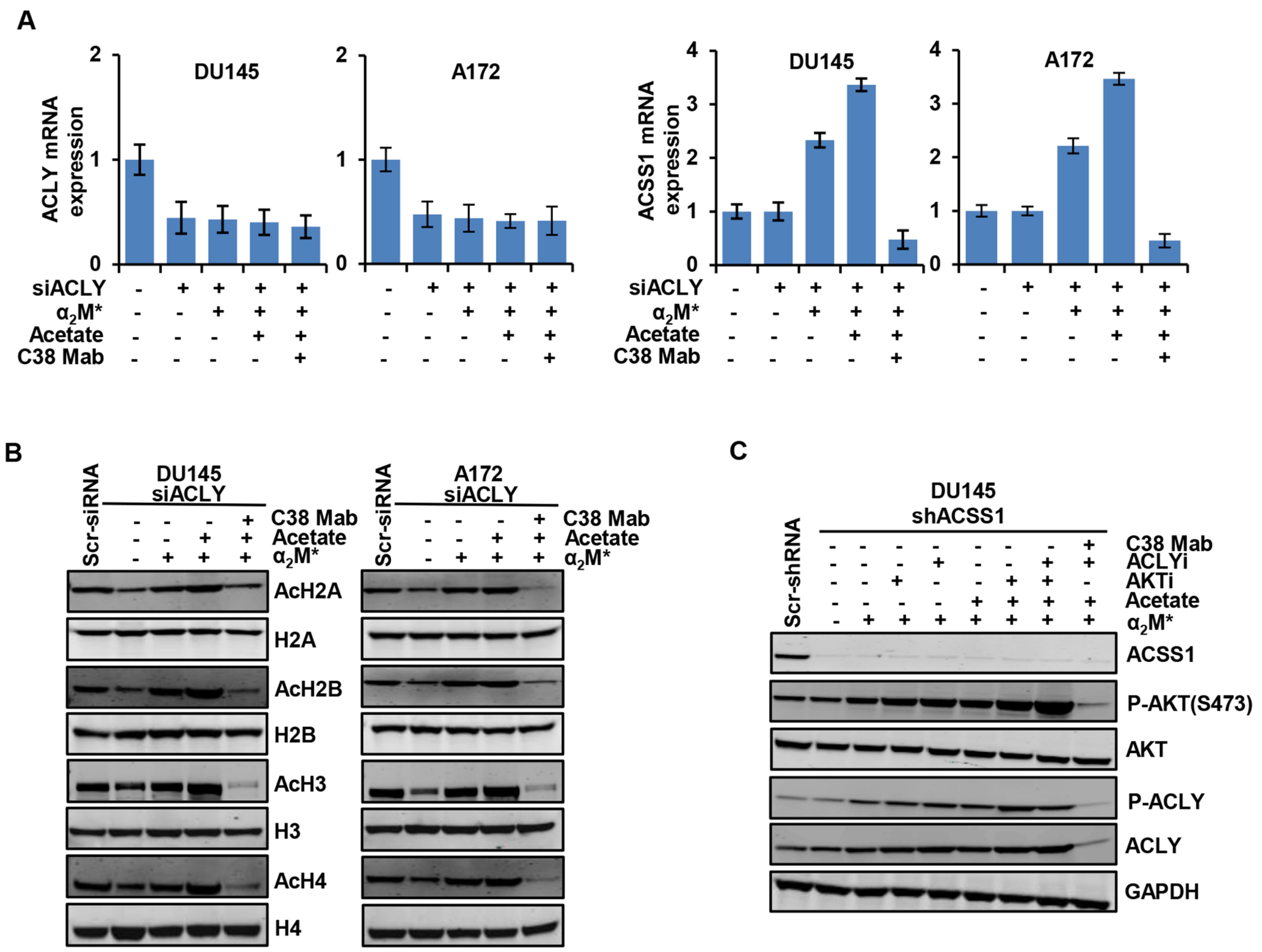

Supplementary Figure 4: α_2M^*/CS-GRP78 regulates both ACLY and ACSS1 expression through AKT pathway. (A) ACLY silencing of indicated cell lines were stimulated either alone or in combination with α_2M^* (100 pM) for 30 min or acetate (5 mM) for 4h in the absence and presence of C38 Mab (50 µg) for 6 h to quantify the transcript level of ACLY and ACSS1genes. **(B)** Immunoblot analysis of histone acetylation levels in ACLY silencing of indicated cells were treated with C38 Mab (50 µg) for 6 h and then stimulated with α_2M^* (100 pM) for 30 min or acetate (5mM) for 4h either alone or in combination. **(C)** Immunoblot analysis of the indicated cancer cell lines stimulated either alone or in combination with α_2M^* (100 pM) for 30 min or acetate (5 mM) for 4h in the absence and presence of ACLYi (100 µM) for 16 h, AKTi (GSK690693, 5 µM/L) for 16 h or C38 Mab (50 µg) for 6 h.

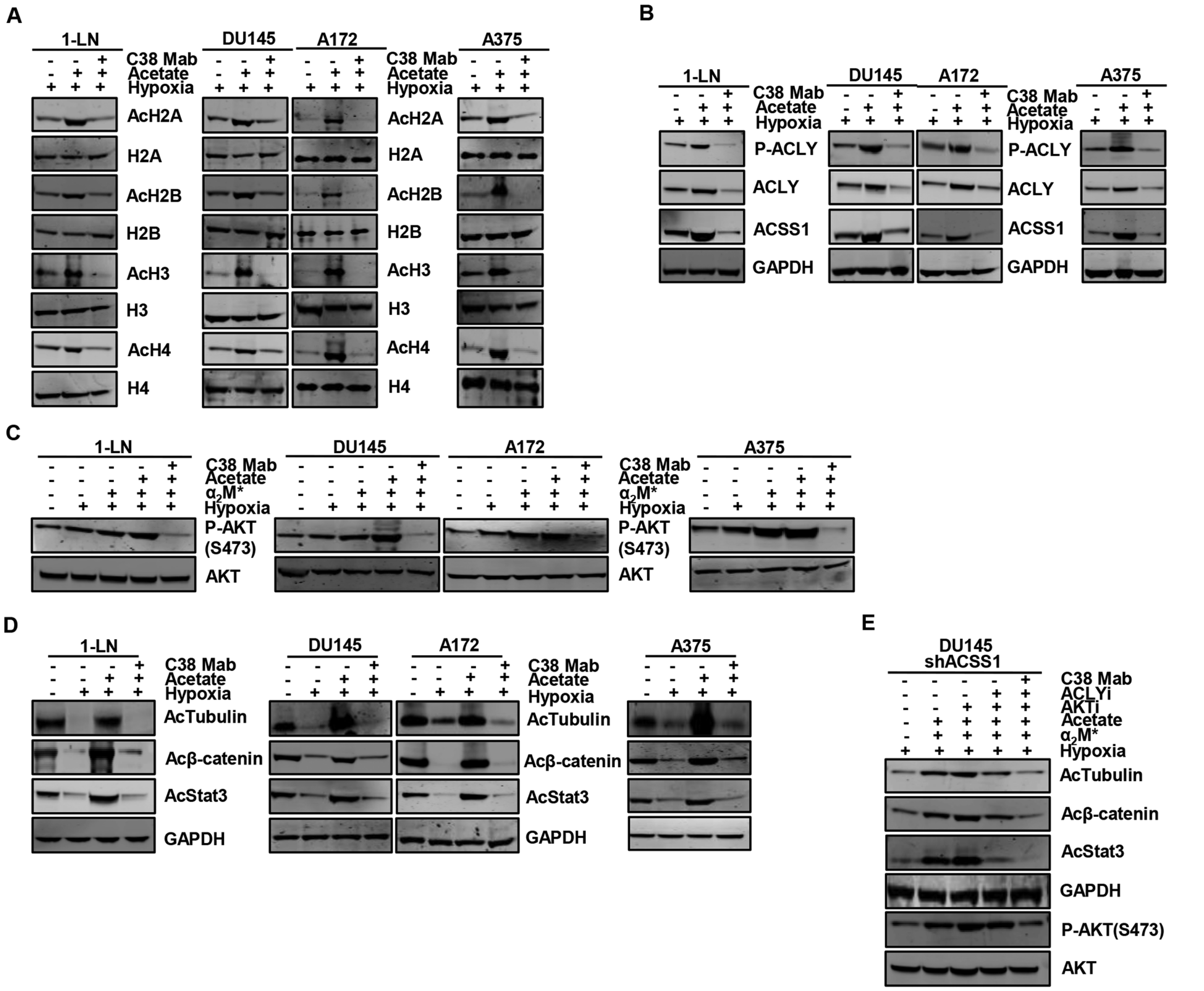

Supplementary Figure 5: Acetate rescues hypoxia induced reduction of histone and protein acetylation in a CS-GRP78 dependent manner. **(A-B)** Immunoblot analysis of histone acetylation in the indicated cancer cell lines under normoxia or hypoxia were treated with C38 Mab (50 µg) for 6 h and then stimulated with acetate (5 mM) for 4h. **(C)** Immunoblot analysis of the indicated cancer cell lines under normoxia or hypoxia treated with C38 Mab (50 µg) for 6 h and then stimulated with α_2M^* (100 pM) for 30 min and acetate (5 mM) for 4h. **(D)** Immunoblot analysis of protein acetylation in the indicated cancer cell lines under normoxia or hypoxia treated with C38 Mab (50 µg) for 6 h and then stimulated with acetate (5 mM) for 4h. **(E)** Immunoblot analysis of protein acetylation levels in ACSS1 silencing of DU145 cells under normoxia or hypoxia stimulated either alone or in combination with α_2M^* (100 pM) for 30 min or acetate (5 mM) for 4h in the absence and presence of ACLYi (100 µM) for 16 h, AKTi (GSK690693, 5 µM/L) for 16 h or C38 Mab (50 µg) for 6 h.

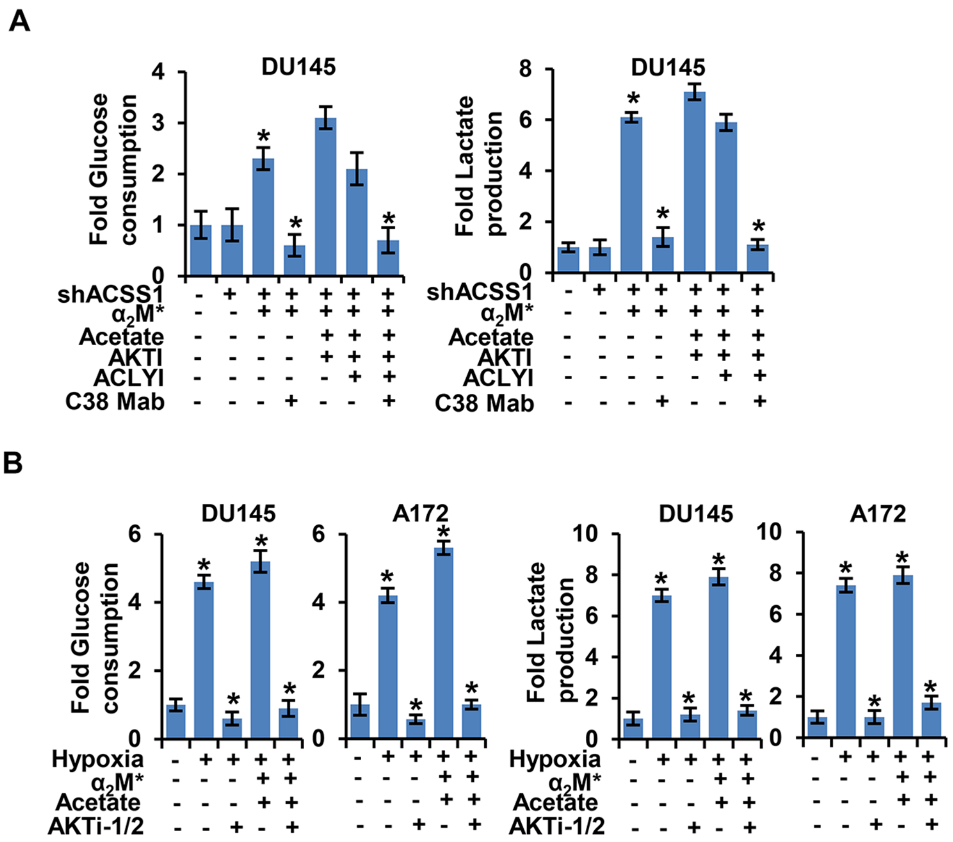

Supplementary Figure 6: α_2M^*/CS-GRP78 regulates Glucose consumption and lactate production. **(A)** Glucose consumption and lactate production were measured in ACSS1 silencing of DU145 cells stimulated either alone or in combination with α_2M^* (100 pM) for 30 min or acetate (5 mM) for 4h in the absence and presence of ACLYi (100 μM) for 16 h, AKTi (GSK690693, 5 μM/L) for 16 h or C38 Mab (50 μg) for 6 h. mean ± SEM of triplicates. *, p values ≤ 0.05. **(B)** Glucose consumption and lactate production were measured in the indicated cancer cell lines under normoxia or hypoxia were stimulated in combination with α_2M^* (100 pM) for 30 min or acetate (5 mM) for 4h in the absence and presence of AKTi-1/2 (10 μM) for 16 h. mean ± SEM of triplicates.
